# Methionine Improves Boar Sperm Quality by Promoting Mitochondrial Translation during Liquid Storage

**DOI:** 10.3390/ani14152227

**Published:** 2024-07-31

**Authors:** Meiling Tan, Yuting Zhao, Li Ren, Chenxuan Li, Jiangxue Cai, Bin He

**Affiliations:** 1 Key Laboratory of Animal Physiology & Biochemistry, Ministry of Agriculture and Rural Affairs, College of Veterinary Medicine, Nanjing Agricultural University, Nanjing 210095, China; 18473448821@163.com (M.T.); zhaoyuting12138@163.com (Y.Z.); 2019107006@njau.edu.cn (L.R.); 2022207005@stu.njau.edu.cn (C.L.); caijsnow@163.com (J.C.); 2 MOE Joint International Research Laboratory of Animal Health & Food Safety, Nanjing Agricultural University, Nanjing 210095, China

**Keywords:** methionine, mitochondrial protein translation, MTFMT, sperm, boar

## Abstract

**Simple Summary:**

Artificial insemination is a common breeding technique employed in the contemporary livestock industry. However, the efficacy of this technique is contingent upon the quality of the sperm utilized. Several factors influence the quality of sperm during liquid storage. It is, therefore, essential to select an effective physiological regulator in order to maintain sperm quality during this process. This study aimed to evaluate the expression of MTFMT in different motile sperm and to investigate the effects of the addition of amino acids and methionine on sperm quality during liquid storage of boar sperm. The results demonstrated that MTFMT was highly expressed in high-motility sperm. Methionine could act as a substrate for protein translation, affecting mitochondrial function by regulating mitochondrial protein translation and directly influencing boar.

**Abstract:**

Boar sperm quality serves as an important indicator of reproductive efficiency, playing a direct role in enhancing the output of livestock production. It has been demonstrated that mitochondrial protein translation is present in sperm and plays a crucial role in regulating sperm motility, capacitation and in vitro fertilization rate. The present study aimed to determine whether methionine supplementation enhances mitochondrial translation in boar sperm, thereby improving sperm quality. The results showed a significant elevation in the abundance of mitochondrial methionyl-tRNA formyltransferase (MTFMT), a crucial enzyme for mitochondrial protein translation, and mitochondrial DNA-encoded cytochrome c oxidase subunit 1 (COX1) in boar sperm exhibiting high motility. Both amino acids and methionine supplementation significantly enhanced boar sperm motility during storage. Moreover, methionine supplementation mitigates the loss of acrosomal integrity, enhances the expression of COX1, and boosts mitochondrial activity. Furthermore, the positive impact of methionine was negated in the presence of the mitochondrial translation inhibitor chloramphenicol. Together, these findings suggest that boar sperm may utilize methionine as a protein translation substrate to enhance sperm motility by stimulating mitochondrial protein translation. The supplementation of methionine may enhance the quality of boar sperm, thereby providing guidance for the optimization of diluent formulations for liquid storage and the identification of physiological regulators that regulate sperm motility.

## 1. Introduction

Artificial insemination (AI) is a common breeding technique used in the livestock industry nowadays and has been used in over 90% of countries globally [1,2]. It is extensively employed on farms to boost the utilization rate of males, efficiently sustain the genetic integrity of superior breeds, limit the dissemination of diseases resulting from inbreeding, alleviate mating difficulties arising from physical disparities, and thereby generate significant economic gains for the livestock [3,4]. Unfortunately, the quality of fresh boar semen deteriorates rapidly, restricting the ability to store it for extended periods, thereby limiting the benefits of utilizing high-quality boars. Sperm are collected and introduced into the isolated reproductive environment of the female animal, where they are highly susceptible to external environmental factors [1]. Therefore, the exploration of novel semen preservation techniques has the potential to broaden the scope of AI in terms of both geographical and temporal constraints.

Mammalian sperm possess a unique protein translation system that utilizes mitochondrial-type ribosomes to translate nuclear-encoded proteins [5,6]. Inhibition of protein translation by chloramphenicol (CP) significantly reduced the levels of 22 proteins, including sperm-specific proteins such as protein kinase C (PKC), angiotensin II type I receptor (AT1-R), and progesterone receptor (PR) [5,6]. These proteins are involved in sperm movement, capacitation, acrosomal reaction, and the fertilization process [6]. Mitochondrial protein translation is initiated with fMet-tRNA^Met^ as the initiating amyl-tRNA but not Met-tRNA. The nuclear-encoded mitochondrial methionyl tRNA methyltransferase (MTFMT) plays a crucial role in mitochondrial protein translation. MTFMT is responsible for the addition of a formyl group to a portion of a single mitochondrial Met-tRNA^Met^, generating fMet-tRNA^Met^ and initiating translation [7,8]. Diniz et al. conducted a genome-wide association study on Large White pigs and identified MTFMT as the sole candidate gene associated with sperm motility [9]. However, the role of mitochondrial protein translation in regulating sperm quality during liquid storage remains elusive.

Methionine plays a crucial role as the initiating amino acid in protein translation and serves as a substrate for the synthesis of Met-tRNA^Met^ [10]. The supplementation of methionine during the cryopreservation of ram sperm enhanced sperm viability and motility and led to a significant increase in the proportion of sperm with high mitochondrial membrane potential (Δψm) [11]. Conversely, mice fed with a methionine- and choline-deficient diet have reduced sperm motility and total number of sperm [12]. The precise function of methionine in regulating sperm quality during liquid preservation remains elusive, as its involvement in mitochondrial protein translation remains unresolved.

Based on these hypotheses, we investigate the utilization of methionine by boar sperm as a substrate for protein synthesis in order to maintain and/or enhance sperm motility through the promotion of mitochondrial protein translation. It could provide insights into enhancing boar semen quality during preservation, emphasizing the potential role of methionine as a physiological regulator in maintaining boar sperm motility during liquid storage.

## 2. Materials and Methods

### 2.1. Sperm Separation

Different motility boar sperm were separated by the Percoll discontinuous density gradient centrifugation method, as described previously [13]. We prepared 90% and 45% Percoll in saline, then added 0.5 mL of 90% Percoll to a 2 mL centrifuge tube, 0.5 mL of 45% Percoll along the wall, and finally added 0.5 mL of dispersed sperm to the top layer, and centrifuged at 650× *g* for 30 min, then removed the layered fluid (top layer of low viability sperm and bottom layer of high viability sperm) and added an equal volume of saline. Furthermore, we removed the stratified fluid (the upper layer of low motility sperm and the lower layer of high motility sperm), added an equal volume of saline, centrifuged at 430× *g* for 10 min, removed the supernatant, and resuspended the sperm with the appropriate amount of saline to elute the sperm. The separated sperm with high and low motility were collected and set aside.

### 2.2. Amino Acids and Methionine Supplementation

To investigate the effects of amino acids supplementation on boar sperm motility, boar semen was randomly allocated into four groups: the control group, the amino acid group, which was prepared by diluting the total amino acids to a final concentration of 1×, using 100× non-essential amino acids (Sigma-Aldrich, TMS-001, St. Louis, MO, USA) and 50× essential amino acids (Thermo Fisher, prod#11130051, Waltham, MA, USA), the CP (1.2 μg/mL, Beyotime, ST1150, Shanghai, China) group, the and amino acids + CP (1.2 μg/mL) group, which were incubated at 17 °C for 24 h. To test the effects of methionine (Sigma-Aldrich, M9625-25G, St. Louis, MO, USA) supplementation on boar sperm motility, different concentrations of methionine (0, 0.2, 1 and 5 mM) were added to the semen extender. Then, the semen samples were incubated at 17 °C for 1, 3, and 5 days or incubated at 37 °C for 0, 3, and 6 h. To evaluate the effects of methionine supplementation on sperm motility, mitochondria activity and acrosomal integrity, boar semen was divided into four groups: the control group, the 0.2 mM methionine group, the CP (1.2 μg/mL) group, and the 0.2 mM methionine + CP (1.2 μg/mL) group, incubated at 17 °C for 1, 3, and 5 days.

### 2.3. Evaluation of Sperm Motility

Sperm motility was recorded using a sperm motility analyzer (Sperm Vision 3.5, Minitube, Germany) [14]. The incubated sperm were gently blown and mixed with a pipette, 10–20 μL was added dropwise to the pre-warmed slides and the coverslips were carefully placed on a thermostatic slide. The images were adjusted and analyzed using Sperm Vision 3.5 software.

### 2.4. Protein Extraction and Western Blot Analysis

Total proteins were extracted from sperm under different treatments with lysis buffer (Bioworld, BD0032, Bloomington, IN, USA). The protein concentration was measured with Pierce, a BCA Protein Quantification Kit (Thermo Fisher, prod#23227, Waltham, MA, USA). The proteins were then separated by 10% SDS-PAGE and transferred to a PVDF membrane. The membrane was blocked with 5% skimmed milk for 2 h at room temperature and then incubated with primary antibody MTFMT ( Cloud-Clone Corp, 1:1000, PHA614Mu01,Houston, TX, USA), COX1 (Bioworld, 1:1000, BS1636, Bloomington, IN, USA), and β-actin (Bioworld, 1:2000, BS6007M, Bloomington, IN, USA) at 4 °C overnight. The rabbit anti-rabbit (1:10,000 final dilutions) was used as a secondary antibody and incubated at room temperature for 2 h. The ECL Plus Western blotting detection system (Tanon Science & Technology, Tanon-5200, Shanghai, China) was used to detect the band intensity. Finally, ImageJ 1.54j was used to quantify the relative signal intensities of the bands [15].

### 2.5. Mitochondrial Membrane Potential (Δψm) Analysis

Mitochondrial membrane potential variations, Δψm, were evaluated using a specific probe JC-1 (Thermo Fisher, T3168, Waltham, MA, USA) [13]. This lipophilic cationic fluorochrome JC-1 is present as protomeric aggregates in mitochondria with high membrane potential that emit at 590 nm when excited at 549 nm, whereas in mitochondria with low membrane potential, JC-1 is present as monomers that emit at 525 nm when excited at 488 nm. Briefly, an aliquot of 100 mL from each sperm sample (1 × 10^6^ cells/mL) was diluted in 400 mL of isotonic buffer containing 0.15 mmol/L of JC-1 and then mixed and incubated at 37 °C for 30 min. The samples were mixed again before flow cytometry analysis. The fluorescence was estimated at excitation wavelengths of 488 nm and 549 nm, measured at emission wavelengths of 530 nm and 585 nm, respectively, and 10,000 events were acquired on flow cytometry.

### 2.6. Acrosome Integrity Analysis

We took two clean slides, marked the frosted surface with a pencil, added 10–20 μL of treated sperm on one side of the slide, and placed the other slide on the droplet at an angle of 45°, then slowly pulled the upper slide to the opposite side when the sperm spread to the contact surface until the entire slide below was coated to make a uniform sperm smear. Sperm smears were laid out on a clean paper towel and allowed to dry at room temperature before being submerged in a Coomassie brilliant blue (Shanghai yuanye Bio-Technology CO., Ltd, S19061, Shanghai, China) stain for 1.3 h. Excess stain was carefully washed off with triple distilled water, dried, observed under a microscope, and photographed. Each smear was photographed in 8 different fields of view, and the same smear was counted by 3 individuals [16].

### 2.7. Statistical Analysis

The experimental data were expressed as mean ± SEM and were statistically calculated using SPSS 26 software (IBMCorp., Armonk, NY, USA) and compared using an independent samples *t*-test, one-way ANOVA, and two-way ANOVA. The differences were considered statistically significant when *p* < 0.05.

## 3. Results

### 3.1. The Abundance of MTFMT and COX1 in Different Motility Sperm

**Figure 1:**
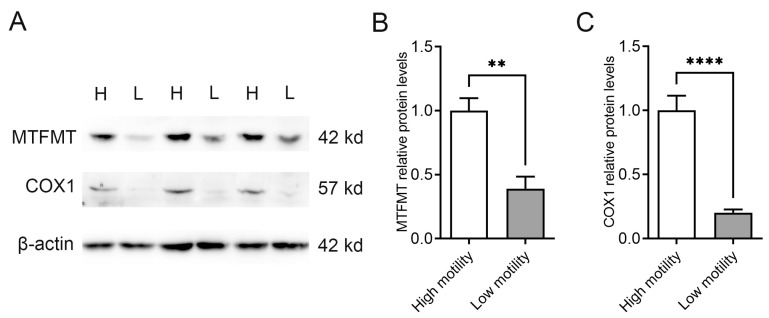
The abundance of MTFMT in different motility sperm. (**A**) Western blot analysis of MTFMT and COX1 in boar sperm. (**B**) Quantification of MTFMT in boar sperm. (**C**) Quantification of COX1 in boar sperm. Student’s *t*-test was performed to analyze the difference between MTFMT and COX1 abundance (**B**,**C**). Values are expressed as mean ± SEM, n = 6. ** *p* < 0.01; **** *p* < 0.0001. H: high motility; L: low motility.

The MTFMT functions in the early stages of mitochondrial translation. Different motility boar sperm were separated by Percoll discontinuous density gradient centrifugation, and the abundance of MTFMT in different motility sperm was detected by Western blot. The results showed that the abundance of MTFMT protein was significantly higher in high-motility sperm than in low-motility sperm (Figure 1A,B). Furthermore, cytochrome c oxidase subunit 1 (COX1), encoded by mtDNA, exhibited significantly higher levels in high-motility sperm compared with those in low-motility sperm (Figure 1A,C).

### 3.2. Effects of Amino Acids Supplementation on Boar Sperm Motility

To assess the potential of amino acids supplementation in improving sperm motility, amino acids essential for protein synthesis were incorporated into the semen extender and incubated at 17 °C for 24 h. The results showed that amino acids supplementation have a notable impact on enhancing the proportion of sperm with progressive motility (Table 1). Conversely, CP, which inhibits mitochondrial protein translation, effectively prevented the increase in the proportion of sperm with progressive motility (Table 1). These findings suggest that amino acids may improve sperm motility by boosting mitochondrial protein translation.

### 3.3. Effects of Methionine Supplementation on Sperm Motility

Methionine serves as both the initial amino acid in protein translation and as a substrate for MTFMT. To determine the potential impact of methionine on boar sperm quality, we incorporated varying concentrations of methionine (0.2, 1, 2 mM) into the extender for 5 days at 17 °C. The results showed that sperm motility was significantly increased with 0.2 mM and 1 mM methionine supplementation compared with the control group on the third day. Additionally, the percentage of progressively motile sperm in the 0.2 mM methionine supplementation group was significantly higher than the control group on the third day. Sperm motility and progressive motility in the 0.2 mM methionine supplementation exhibited a rising trend, although not statistically significant on the fifth day (Table 2).

Subsequently, CP was introduced to examine the involvement of mitochondrial translation in methionine-induced enhancement of sperm motility. The promotional effect of methionine on sperm motility and progressive motility was found to be significantly suppressed by CP (Table 3). These findings suggest that methionine may improve sperm motility by boosting mitochondrial protein translation.

We then evaluated the effect of methionine supplementation on sperm incubated for 3 and 6 h at 37 °C. The sperm motility showed a significant increase in the 0.2 mM methionine group after 3 h incubation, while progressive motility exhibited a rising trend, although not statistically significant (Table 4).

### 3.4. Effects of Methionine Supplementation on Sperm Mitochondria Activity

**Figure 2:**
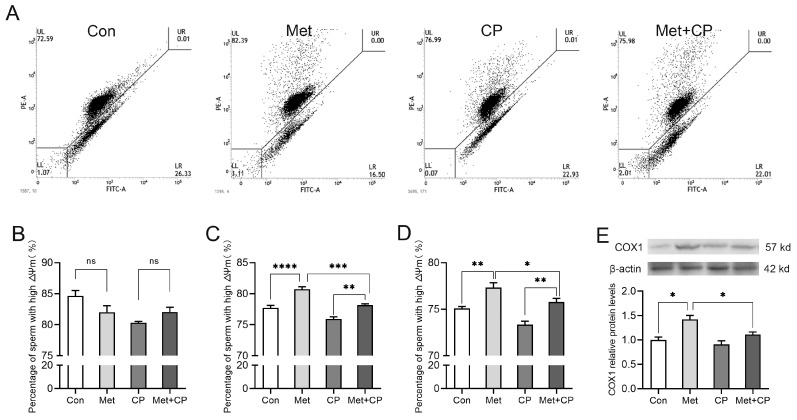
Effects of methionine on sperm motility and mitochondria activity. (**A**) Detection of sperm mitochondrial membrane potential by flow cytometry. (**B**–**D**) A percentage of sperm with high membrane potential was stored with 0.2 mM methionine and 1.2 μg/mL CP for 1 d (**B**), 3 d (**C**), and 5 d (**D**). (**E**) Western blotting analysis of COX1. Con: control group; Met: 0.2 mM methionine treatment group; CP: 1.2 μg/mL chloramphenicol treatment group; CP + Met: 1.2 μg/mL chloramphenicol and 0.2 mM methionine treatment simultaneously group. One-way ANOVA was performed to analyze the effect of Met on sperm mitochondrial membrane potential (**B**–**D**). Student’s *t*-test was performed to analyze the difference of COX1 abundance (**E**). Values are expressed as mean ± SEM, n = 6. * *p* < 0.05; ** *p* < 0.01; *** *p* < 0.001; **** *p* < 0.0001.

Mitochondrial activity serves as a crucial viability indicator in sperm and is intricately linked to sperm motility. To investigate the impact of methionine on sperm mitochondrial activity during storage, 0.2 mM methionine and CP were added to the extender and stored at 17 °C for 5 d. There was no significant difference in the percentage of sperm exhibiting high Δψm among the groups on the initial day of storage (Figure 2A,B). On the third and fifth days, there is a significant increase in the percentage of sperm with high Δψm in response to methionine supplementation (Figure 2A,C,D). Compared with the methionine supplementation group, the presence of CP led to a decrease in Δψm in sperm on the third and fifth days (Figure 2A,C,D). Western blot analysis demonstrated a significant increase in the abundance of COX1, encoded by mtDNA, in the methionine-supplemented group versus the control group. This increase was attenuated by CP treatment (Figure 2E).

### 3.5. Effects of Methionine Supplementation on Acrosomal Integrity

The acrosomal integrity of the sperm gradually decreased with longer storage times (Table 5). There was no significant difference in the proportion of sperm exhibiting acrosomal integrity between CP and methionine groups following the first and third day of storage at 17 °C. Interestingly, the proportion of sperm exhibiting acrosomal integrity was significantly higher in the methionine group compared with the other groups after storing the semen at 17 °C for the fifth day (Table 5). Furthermore, CP attenuated the stimulatory impact of methionine on sperm acrosomal integrity (Table 5).

## 4. Discussion

The quality of boar sperm serves as a crucial determinant of reproductive efficiency, thereby directly influencing the overall efficiency of livestock production. Here, we found that both amino acids and methionine supplementation significantly enhance boar sperm motility during liquid storage. Moreover, methionine supplementation mitigated the loss of acrosome integrity, enhanced the expression of COX1, and boosted mitochondrial activity during liquid storage. Furthermore, the positive impact of methionine was negated in the presence of the mitochondrial translation inhibitor.

Sperm are collected and introduced into the isolated reproductive environment of the female animal, where they are highly susceptible to external environmental factors [1]. The supplementation of 0.2 mM methionine to extender significantly increased sperm motility and acrosome integrity in boar sperm. It was consistent with the results of Bucak et al. (2012), who demonstrated that methionine supplementation enhances the viability and motility of ram sperm during liquid storage [11]. Furthermore, Jiang et al. reported a significant reduction in testicular and epididymal weights, along with decreased sperm motility and increased sperm malformation, among mice fed a methionine- and choline-deficient diet [12]. A recent study demonstrated that methionine restriction enhances sperm quality in aging mice [17]. Consequently, maintaining an appropriate intake of methionine is paramount for preserving sperm quality.

Mitochondria, located at the central region of sperm, play a crucial role as metabolic organelles. These organelles contain ribosomes, enabling the synthesis of proteins and peptides necessary for the assembly and function of the respiratory chain [18]. Disruption of mitochondrial ribosome assembly has been shown to decrease the expression of OXPHOS proteins and arrest meiosis in spermatocytes [19]. In the present studies, we observed that methionine supplementation enhances sperm motility, accompanied by an increase in Δψm and upregulation of the mitochondrial-encoded protein COX1. This stimulatory effect of methionine on sperm motility was abrogated by a mitochondrial ribosome inhibitor. It was consistent with those reported by Zhu et al. (2019), demonstrating altered sperm motility patterns coincident with modifications in the expression of mitochondrial DNA-encoded genes [20]. Collectively, these results suggest that methionine serves as a substrate for protein translation, ultimately promoting sperm motility through mitochondrial protein synthesis in boar sperm.

A genome-wide association analysis found that MTFMT was the only candidate gene associated with sperm motility [9]. It is a key factor in the translation of mitochondrial proteins encoded by nuclear genes, and its mutations have been described as associated with Leigh syndrome [21]. Our previous research indicated that MTFMT deficiency reduced mitochondrial activity in mice [22]. Here, we found that the expression of MTFMT was higher in high-motility sperm, indicating a potentially crucial role in regulating sperm motility through the promotion of mitochondrial protein translation. Further investigation is required to determine whether methionine serves as a substrate for MTFMT, thereby enhancing sperm motility.

Sperm quality is one of the most important conditions for male fertility and is maintained by many proteins. The acrosome possesses a range of enzymes that enable it to effectively penetrate the zona pellucida during fertilization. It has been demonstrated that mitochondrial translation during capacitation can store proteins beneficial for sperm-egg interaction [23]. ATP1A increased during capacitation in bull sperm, which was due to the presence of mitochondrial translation in mature sperm [24]. Our findings reveal that methionine supplementation mitigated the loss of acrosome integrity during liquid storage. However, the mitochondrial translation inhibitor blocked the stimulatory impact of methionine on maintaining sperm acrosomal integrity. Our findings suggest that methionine preserves acrosome integrity by supporting mitochondrial protein synthesis in boar sperm.

In summary, methionine serves as a substrate for protein translation in boar sperm, thereby enhancing sperm motility via mitochondrial protein synthesis. Furthermore, the inclusion of methionine may improve the quality of boar sperm, thereby guiding the optimization of diluent formulations for liquid preservation. Moreover, it could facilitate the identification of physiological regulators regulating sperm motility.

## 5. Conclusions

In summary, the mitochondrial protein translation pathway plays a critical role in boar sperm. Sperm utilize methionine as a substrate for protein synthesis during translation. The addition of methionine to the diluent during liquid storage will improve the quality of boar sperm.

## Figures and Tables

**Table 1:** The sperm motility and progressive motility of boar semen with amino acids stored at 17 °C for 24 h.

Sperm Motility (%)	Con	AAs	CP	AAs + CP
Total motility	89.50 ± 1.61	93.28 ± 1.20	90.07 ± 83.75	90.00 ± 2.03
Progressive motility	88.03 ± 1.67 ^b^	92.32 ± 1.15 ^a^	88.88 ± 2.43 ^b^	88.47 ± 2.33 ^b^

Con: control, AAs: 0.2 mM amino acids. CP: 1.2 μg/mL chloramphenicol. AAs + CP: 0.2 mM amino acids and 1.2 μg/mL chloramphenicol. Two-way ANOVA was performed to analyze the combined effect of AAs and CP on sperm motility. Columns within rows separated by differing letters are significantly different (*p* < 0.05).

**Table 2:** The sperm motility and progressive motility of boar semen with different doses of methionine stored at 17 °C for 1, 3, and 5 days.

Storage Time	Sperm Motility (%)	Con	Met 0.2	Met 1	Met 5
Day 1	Total motility	89.43 ± 2.34	90.88 ± 1.64	90.53 ± 2.56	90.48 ± 2.38
	Progressive motility	80.81 ± 3.99	81.73 ± 3.51	82.02 ± 4.75	82.27 ± 4.2
Day 3	Total motility	86.86 ± 1.41 ^b^	90.41 ± 1.16 ^a^	89.94 ± 1.42 ^ab^	87.27 ± 2.38 ^ab^
	Progressive motility	77.13 ± 2.44 ^b^	81.42 ± 1.99 ^a^	80.38 ± 2.59 ^ab^	77.63 ± 3.4 ^ab^
Day 5	Total motility	84.0 3± 2.34 ^ab^	85.73 ± 1.73 ^a^	84.67 ± 2.31 ^ab^	79.64 ± 3.51 ^bc^
	Progressive motility	73.09 ± 3.10 ^ab^	75.3 ± 2.44 ^a^	73.54 ± 3.44 ^ab^	67.77 ± 4.79 ^bc^

Con: control, Met 0.2: 0.2 mM methionine, Met 1: 1 mM methionine, Met 5: 5 mM methionine. One-way ANOVA was performed to analyze the effect of Met on sperm motility. Columns within rows separated by differing letters are significantly different (*p* < 0.05).

**Table 3:** The sperm motility and progressive motility of boar sperm supplemented with methionine and chloramphenicol stored at 17 °C for 1, 3, and 5 days.

Storage Time	Sperm Motility (%)	Con	Met	CP	Met + CP
Day 1	Total motility	92.50 ± 2.61	93.28 ± 3.20	91.07 ± 3.75	90.08 ± 2.03
	Progressive motility	83.56 ± 1.99	84.73 ± 2.64	82.02 ± 1.75	82.27 ± 2.12
Day 3	Total motility	87.75 ± 0.92 ^b^	89.23 ± 0.64 ^a^	86.37 ± 0.63 ^ab^	88.03 ± 0.58 ^ab^
	Progressive motility	78.12 ± 1.56 ^b^	83.41 ± 1.37 ^a^	77.51 ± 1.79 ^b^	75.13 ± 1.78 ^b^
Day 5	Total motility	84.52 ± 3.65 ^ab^	88.24 ± 1.27 ^a^	81.07 ± 3.28 ^b^	82.54 ± 3.54 ^b^
	Progressive motility	75.23 ± 2.10 ^ab^	80.30 ± 2.44 ^a^	73.78 ± 1.44 ^bc^	70.17 ± 1.79 ^c^

Con: control, Met: 0.2 mM methionine, CP: 1.2 μg/mL chloramphenicol. Met + CP: 0.2 mM methionine and 1.2 μg/mL chloramphenicol. Two-way ANOVA was performed to analyze the combined effect of AAs and CP on sperm motility. Columns within rows separated by differing letters are significantly different (*p* < 0.05).

**Table 4:** The sperm motility and progressive motility of boar semen with different doses of methionine stored at 37 °C for 3 and 6 h.

Storage Time	Sperm Motility (%)	Con	Met 0.2	Met 1	Met 5
3 h	Total motility	87.25 ± 2.16 ^b^	90.64 ± 1.78 ^a^	87.83 ± 2.25 ^b^	86.29 ± 2.32 ^b^
	Progressive motility	76.18 ± 4.44 ^ab^	79.54 ± 4.45 ^a^	75.18 ± 5.32 ^ab^	69.92 ± 5.89 ^b^
6 h	Total motility	83.34 ± 2.35 ^a^	82.66 ± 3.09 ^ab^	80.09 ± 3.66 ^ab^	73.98 ± 5.14 ^bc^
	Progressive motility	66.11 ± 5.34 ^a^	65.08 ± 6.11 ^a^	60.61 ± 6.56 ^ab^	53.66 ± 6.85 ^b^

Con: control, Met 0.2: 0.2 mM methionine, Met 1: 1 mM methionine, Met 5: 5 mM methionine. One-way ANOVA was performed to analyze the effect of Met on sperm motility. Columns within rows separated by differing letters are significantly different (*p* < 0.05).

**Table 5:** Percentage of sperm with acrosome integrity in boar sperm supplemented with CP and methionine for 1, 3, and 5 days.

Storage Time	Con	Met	CP	Met + CP
Day 1	92.50 ± 2.61 ^a^	93.28 ± 3.20 ^a^	91.07 ± 3.75 ^a^	90.08 ± 2.03 ^a^
Day 3	87.75 ± 0.92 ^a^	86.23 ± 0.64 ^b^	86.37 ± 0.63 ^b^	88.03 ± 0.58 ^a^
Day 5	84.52 ± 3.65 ^Bb^	88.24 ± 1.27 ^Ab^	81.07 ± 3.28 ^Bb^	82.54 ± 3.54 ^Bb^

Con: control, Met: 0.2 mM methionine, CP: 1.2 μg/mL chloramphenicol. Met + CP: 0.2 mM methionine and 1.2 μg/mL chloramphenicol. One-way ANOVA was performed to analyze the effect of Met on sperm motility. The different uppercase letter in the same row indicates significant differences (*p* < 0.05), and the different lowercase letters in the same column indicate significant differences (*p* < 0.05).

## Data Availability

The raw data supporting the conclusions of this article will be made available by the authors upon request.
